# Management of an infectious complication appearing in a transcanine implant: a case report

**DOI:** 10.1186/s40729-025-00626-6

**Published:** 2025-05-19

**Authors:** Maxime Delarue, Pierre Klienkoff, Mélanie Le Ven, Fabien Bornert

**Affiliations:** 1 Faculty of Dental Surgery, University of Strasbourg, 8 Rue de Sainte Elisabeth, 67000 Strasbourg, France; 2 Department of Oral Surgery, UF8601, University Hospital of Strasbourg, 1 Place de L’Hôpital, 67000 Strasbourg, France; 3 Department of Dento-Facial Orthopedics, UF8602, University Hospital of Strasbourg, 1 Place de L’Hôpital, 67000 Strasbourg, France; 4 Dental Care Unit, UF8611, University Hospital of Strasbourg, Hôpital de Hautepierre, 1 Avenue Molière, 67098 Strasbourg, France; 5 INSERM (French National Institute of Health and Medical Research) UMR 1260, Regenerative Nanomedicine, CRBS, 1 Rue Eugène Boeckel, 67000 Strasbourg, France

**Keywords:** Dental implant, Canine, Coronectomy, Transcanine, Impacted tooth, Ankylosis, Decoronation

## Abstract

**Background:**

Maxillary canine impaction is the second most common dental eruption anomaly, affecting approximately 0.2–3% of individuals, with a higher incidence in females. This condition often results in complications such as the misalignment of adjacent teeth, root resorption, and the development of cystic lesions. In some cases, abstention is recommended for impacted canine is kept with the lacteal tooth held on the dental arch. But in the longer term an implant therapy is nevertheless indicated.

**Case presentation:**

A 42-year-old man presented with persistent swelling and pain in the maxillary region associated with a transcanine implant placed one year ago by his dental practitioner. Imaging assessment showed the implant’s apex inserted into the impacted canine which presented a crown and root resorption and was associated to a radiolucency around. In order to preserve implant and reduce morbidity related to a full extraction of the tooth, a coronectomy was performed allowing inflammatory surrounding tissues curettage.

**Discussion:**

This case shows an infectious complication of a transcanine implant and demonstrates an approach for managing these complications while preserving this implant. The coronectomy is a less invasive technique that reduces potential surgical complications and supports healing. A 2-year follow-up revealed complete bone reossification reinforcing the effectiveness of this method in similar clinical scenarios.

**Conclusion:**

This case suggests that coronectomy may be a viable option for managing impacted canines in proximity to implants when complete extraction poses a high risk of complications. However, given the limited number of reported cases and the absence of long-term data, this approach should be considered with caution. Further studies are necessary to better define the indications, long-term outcomes, and potential risks of this technique.

## Introduction

The maxillary canine is the second most frequently impacted tooth, with an incidence rate of 0.2% to 3% in the general population [1]. It occurs when a tooth remains embedded within the oral mucosa or intraosseous structures beyond its expected eruption time. They are more commonly impacted on the palatal side in a 2:1 ratio. This condition is more prevalent in females than in males [2]. Canine impaction can lead to several complications, including migration of adjacent teeth, loss of arch length, and, in some cases, root resorption of the nearby lateral incisors and first premolar [3]. Additionally, impacted canines carry an increased risk of developing cystic lesions and infections [2].

Given the canine’s pivotal role in smile aesthetics, phonation, and masticatory function, the importance of the maxillary canine is paramount. Various approaches are proposed for managing impacted maxillary canine, including interceptive treatments, space creation, orthodontic traction, auto-transplantation and extraction. The choice of therapeutic approach depends on specific criteria and the patient's age. Extraction is typically recommended when other treatment options are not viable, particularly in cases of deeply displaced or severely dilacerated teeth, closed apices or ankylosed teeth [4].

Adult patients seek restorative solutions like fixed prosthetics or dental implant to replace a failing deciduous canine or an edentulous space due to an unerupted adult canine [5]. Implant strategies can be a challenging clinical scenario without evidence-based treatment. Options include extraction of the impacted canine followed by grafting and implant placement, extraction with immediate implant placement, using tilted implants to avoid the impacted canine, placement of short implants and placement of implants through or partially engaging the impacted tooth [6].

In 2009, an unconventional protocol was published in which implants were deliberately placed in contact with dental tissues of impacted canines. The aim was to avoid an invasive extraction when an impacted tooth was found on the ideal way of a planned implant [7]. In 2015, a case series showed nine patients were treated by 12 implants placed through the impacted teeth. All implants were restored, and no implant failed during the 1- to 8-year follow-up [8]. In the literature, different authors have hypothesized that the contact between implant and dental tissues is compatible [9–11]. The concept of mineral integration in trans-corono-radicular implants challenges the traditional paradigm of osseointegration, which relies on direct bone-implant contact. Histological studies suggest that implant-dental interfaces can develop specific adaptations without necessarily compromising stability or biocompatibility. The formation of neo-cement and ligamentous structures in continuity with the periodontal ligament has been observed [12]. Strong adhesion of an apical root fragment to an implant, along with a continuous layer of neo-cement, has also been reported [13]. The presence of mineralized tissue at the implant-root interface has been confirmed [9], while the formation of cementum and ligament-like structures on titanium implants in root environments has been demonstrated [14]. A pulp-driven reparative process has also been highlighted, leading to the formation of osteodentin or osteocementum at the implant interface [15]. Other authors argued that this contact may lead to complications such as infection or failure in the transcanine implant strategy, but any complication was referred [16, 17].

In the present report, we introduce the management, by a minimally invasive approach, of an infectious complication with an implant placed through an upper left maxillary impacted canine.

## Case presentation

### Patient presentation and initial symptoms

A 42-year-old man consulted the Department of Oral Surgery, University Hospitals of Strasbourg, concerning a persistent maxillary painful gingival swelling. He had complaints since few weeks in the gum area of the implant 23 which was placed one year ago by his dental practitioner.

### Clinical and radiological assessment

**Fig. 1:**
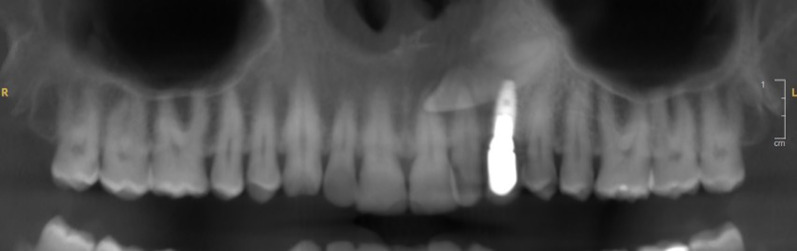
Reconstructed panoramic view of pre-operative CBCT assessment. This view is showing transcanine implant in the tooth 23

**Fig. 2:**
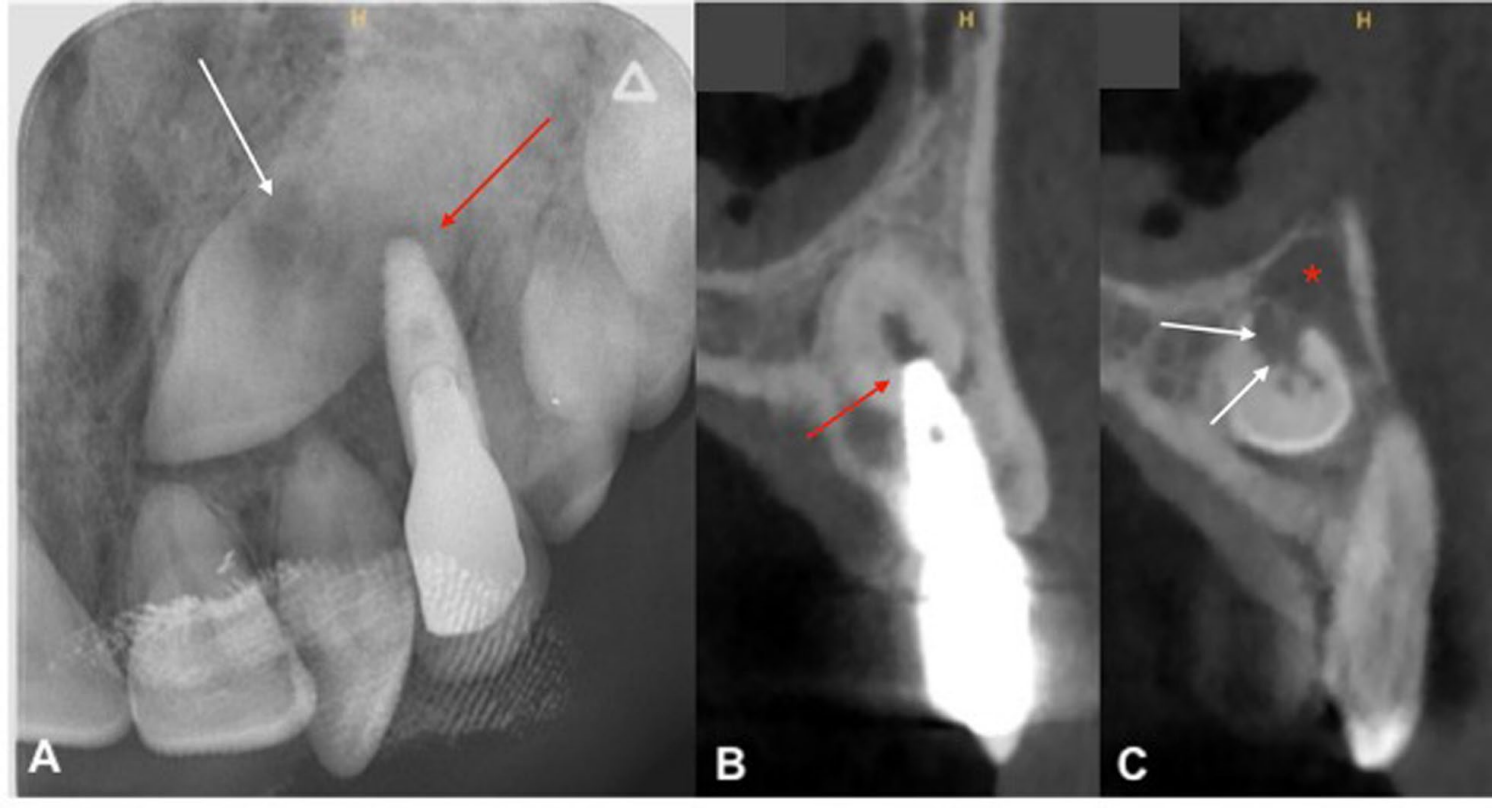
Preoperative radiographic view. **A** Maxillary occlusal radiograph. **B** Orthoradial slice of maxillary through tooth 23. **C** Orthoradial slice of maxillary through tooth 22. Red arrows are showing insertion of implant apex inside the tooth. White arrows are showing internal–external resorption and red star is showing radiolucency

The sensitivity dental pulp test was normal on teeth 21 and 22. Axial and transverse percussion test was normal on implant 23. The orthopantomogram (OPG) revealed the presence of a horizontally impacted maxillary canine (tooth 23) in contact with the apical portion of implant (Fig. 1). An occlusal X-ray showed an internal–external resorption of the cervical portion of impacted tooth 23 (Fig. 2A).

**Fig. 3:**
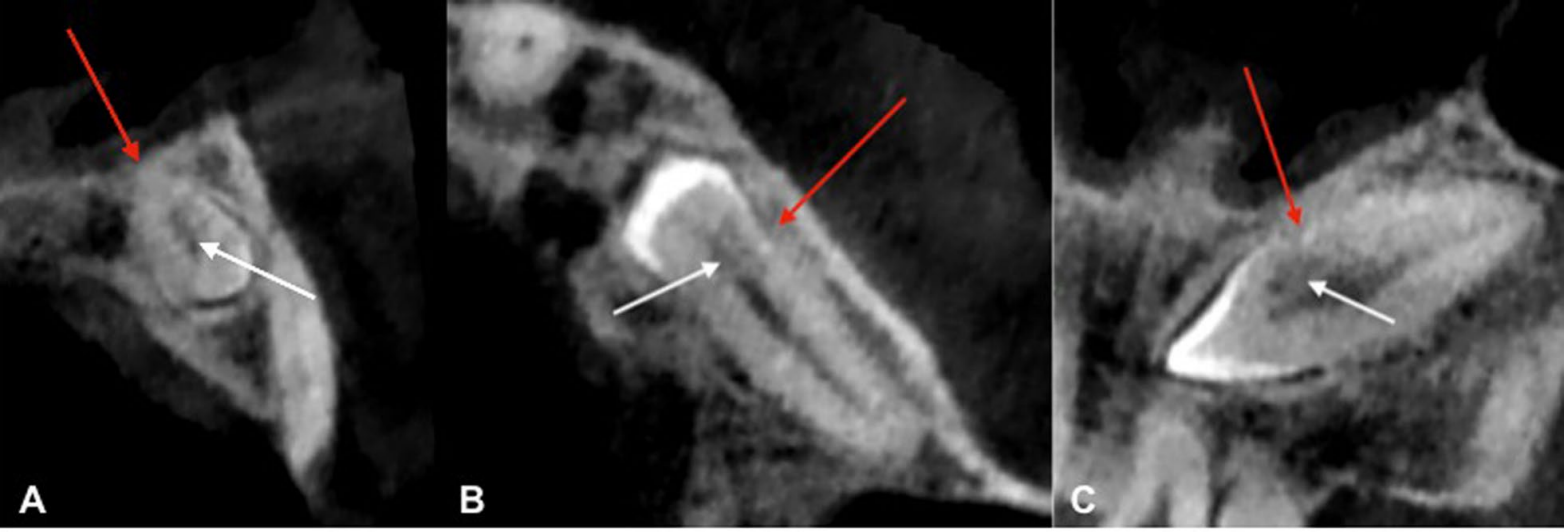
CBCT views before transcanine implant placement. **A** Orthoradial slice of maxillary through tooth 23. **B** Axial slice of maxillary through tooth 23. **C** Frontal slice of maxillary through tooth 23. Red arrows are showing signs of ankylosis of cervical portion and white arrow are showing internal resorption

A Cone Beam Computed Tomography (CBCT) confirmed the implant tip was through the crown third of the tooth in close contact with the dental pulp (Fig. 2B). The tooth 23 presented a radiolucency (9 × 7 × 11 mm) in contact with the upper part of the crown in continuity of the resorption area and extending buccally and anteriorly (Fig. 2C). This radiolucency was also into contact with the anterior portion of the left nasal of the floor. This radiolucency lesion was not visible on CBCT performed before transcanine implant placement. There were nevertheless some discreet signs of internal resorption visible on this CBCT (Fig. 3A–C).

All these elements suggested an inflammatory pericoronal lesion in relation with internal tooth resorption induced by dental implant with subsequent infectious complication [18].

### Treatment plan and preoperative management

A surgical approach was proposed. Nonetheless a curative antibiotic therapy was prescribed to treat the dental infection before surgical procedure. The primary risks associated with the extraction of the impacted maxillary canine (tooth 23) included potential loss of the implant 23, loss of vitality of teeth 21 and 22, the development of a significant bone defect, and a potential perforation of the nasal floor. We opted for an unconventional approach which involved performing a coronectomy of the impacted maxillary canine combined with the enucleation of the inflammatory lesion and preservation of dental implant.

The preoperative medications included oral amoxicillin at a dose of 1 g 1 h before surgery, followed by 1 g three times daily for seven days. For pain management, oral acetaminophen was administered at 1 g every six hours, with a maximum duration of seven days. Additionally, a single morning dose of 60 mg of corticosteroids was prescribed, starting on the morning of surgery and continuing for three days to reduce inflammation and postoperative swelling. A chlorhexidine mouthwash (0.12%) was also recommended, to be used 48 h prior to surgery and continued 48 h post-surgery, for a total of seven days, to ensure optimal oral hygiene and reduce the risk of post-operative infection.

### Surgical procedure

**Fig. 4:**
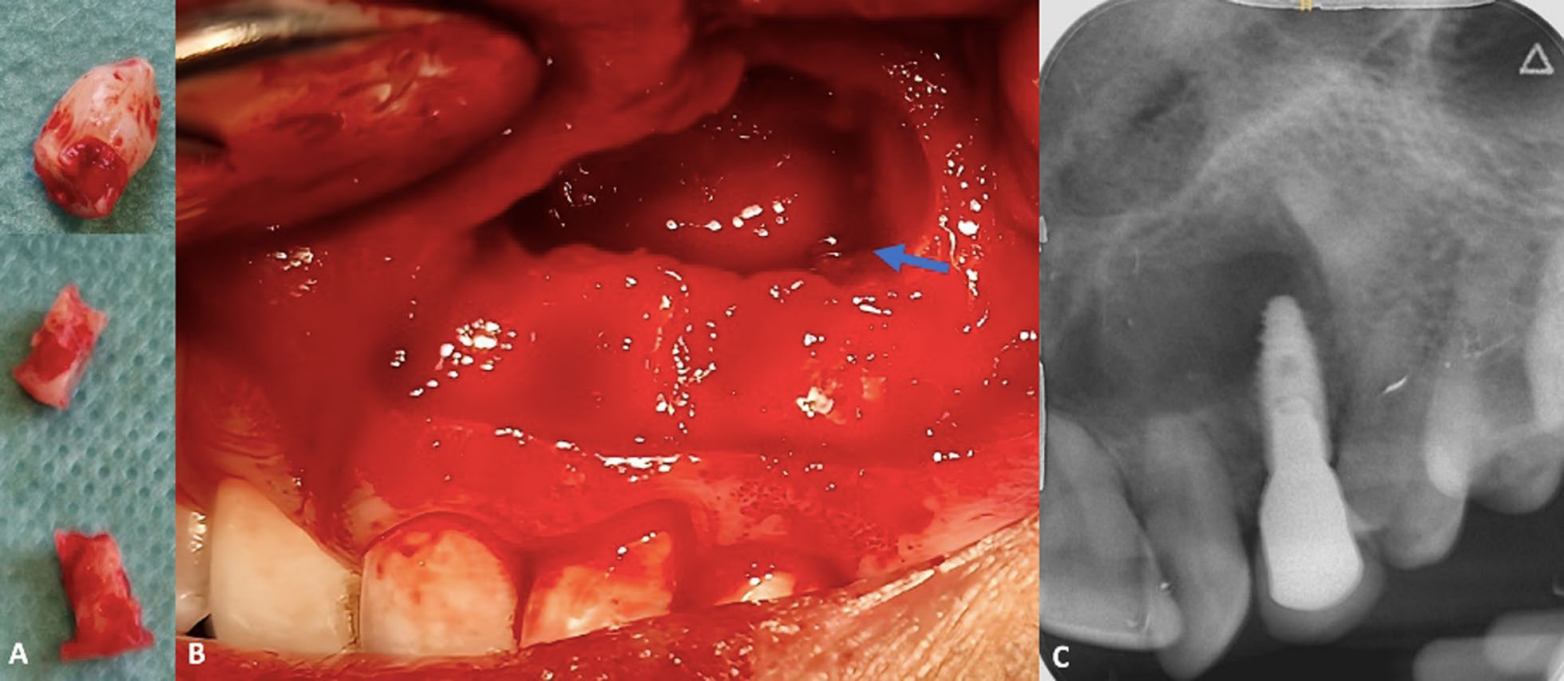
Clinical views of surgical procedure. **A** Extracted fragments with the presence of the crown and mesial and distal root sections of implant 23. **B** Intraoral operative view showing the sectioning of the crown of the impacted canine and visualization of the apical portion of implant 23 (blue arrow), distant from the residual root. **C** Per operative maxillary occlusal radiograph confirms the complete removal of enamel before suturing

Under local anesthesia, a surgical buccal exposure was achieved through a paramarginal incision with complementary distal and mesial incisions were made to release a full-thickness flap. The impacted canine crown was exposed via an ostectomy with a round bur. Coronectomy was achieved using a red contra-angle and fissure bur (H162SL.314.014 VPE 1 tungsten bur, Komet, Dublin, Ireland). The impacted maxillary crown was extracted in several fragments from the mesial and distal sides of the implant (Fig. 4A). The ankylosis of the root portion was confirmed surgically by a lack of mobility. Then, cyst enucleation and surgical site revision was performed (Fig. 4B) and submitted for histopathological examination. An occlusal radiograph was performed to confirm the absence of enamel residue (Fig. 4C). An endodontic file (K file, size 15) was used to remove the remaining pulp parenchyma from the apical fragment, inducing intracanal bleeding and promoting supra-radicular blood clot formation. The bone walls were curetted until the cavity was fully filled by a blood clot. A pericardium collagen membrane 15 × 20 mm (Jason^®^ membrane, botiss biomaterials GmbH, Zossen, Germany) was placed on the ostectomy area to prevent tissue invagination and promote bone healing. The wound was closed by single stitches (5–0, Vicryl Rapide, Raritan, NJ, USA).

### Histopathological findings

The histological analysis revealed fragments of a fibrous cyst wall lined with non-keratinizing stratified squamous epithelium. The cyst wall showed focal areas of mild chronic inflammation, predominantly composed of lymphocytes and plasma cells, with occasional neutrophils. Fragments of vital bone were also present, and stromal cholesterol clefts were noted.

In conjunction with the clinical history and radiographic findings, these histological features confirmed the diagnosis of a pericoronal inflammatory cyst associated to invasive tooth resorption induced by dental implant trauma [18].

### Postoperative outcome and follow-up

**Fig. 5:**
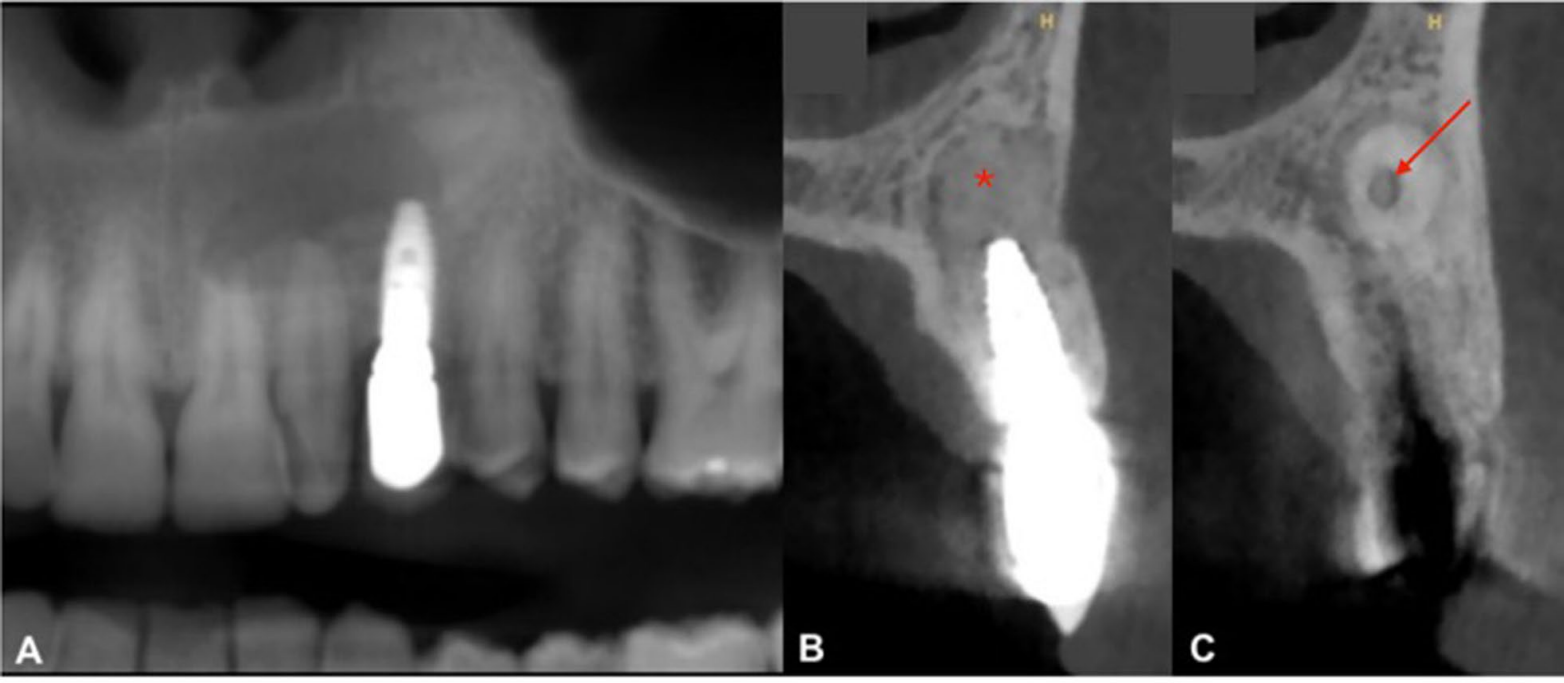
CBCT views with follow-up at 8 months. **A** Reconstructed panoramic view. **B** Orthoradial slice of maxillary through implant 23. **C** Orthoradial slice of maxillary through residual apical root tooth 23. This view’s showing complete reossification of the bony defect around implant 23

**Fig. 6:**
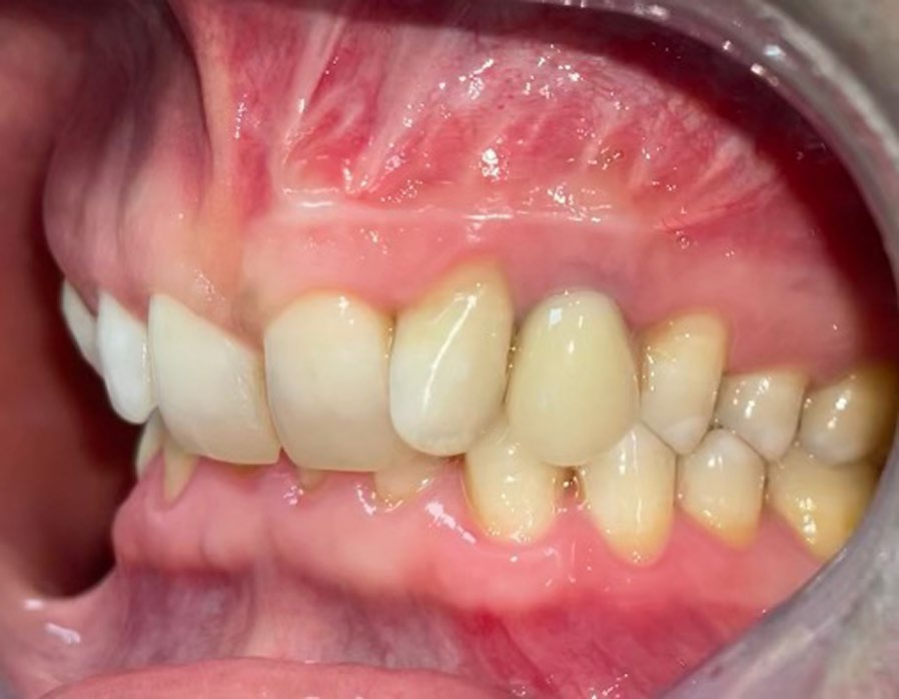
Intraoral view at 2 years of follow-up

Soft tissue healing was complete after two weeks. After 8 months of follow-up a complete reossification of the bony defect occurred in the cavity and in the apical area of the implant 23 (Fig. 5A–C). After 2 years of follow-up, no local recurrence was noted and maintaining the integrity of teeth 21, 22 and implant 23 (Fig. 6).

## Discussion

This case report is the first dedicated to the complication management of an infected tooth resorption in a transcanine implant, as detailed in Table 1 [8, 19–25]. The only complications reported in the literature include the loss of integration of an 8.5 mm short implant within four months of placement in a maxillary impacted canine [26], and bone loss with exposed threads associated with slight mobility one month after the placement of an implant in a mandibular impacted canine [25].

**Table 1:** Details of implants placed through impacted canines from the literature: characteristics and clinical outcomes

First author/year	Study type	Cohort	Age (y)	Sex	Indication	Impacted canine	Implant number (n = 30)	Implant type (Brand, diameter, length)	Surgical process	Crossed the pulp chamber (yes/no)	Access (radicular vs. coronal)	Implant apex position	Implant Healing	Prosthetic and biological complication (yes/no)	Duration before prosthesis (months)	Survival Rate before prosthesis	Follow-up (y)	Success Rate after prosthesis
Davarpanah [19] 2009 + Mithridade [20] 2015^a^	Case series	1	62	F	Complete maxillary rehabilitation	13, 23	3	13: Osséotite^®^ 3.75*11.5 23: Osséotite^®^3.75*8.5 24: Osséotite^®^ XP 4/5*15	Conventional drilling	Ns	3 Radicular	Tooth Tooth Bone	Two-stage	Yes Mobility 24 after 4 months Replaced after canine removal	12	3/4 (75%)	4	3/3 (100%)
		1	31^a^	F	Canine edentulism	13	1	NT Osséotite^®^ 5*15	Oversized drilling 1/2 coronal portion	Yes	Coronal	Bone	Two-stage	No	6		8	
Szmukler- Moncler [21] 2014	Case report	1	64	F	Canine edentulism	13	1	Nobel Active^®^ 4.3*13	Guided Surgery	Yes	Coronal	Bone	One-stage	Yes Hematoma right cheek	6	1/1 (100%)	1.5	1/1 (100%)
Davarpanah [8] 2015	Case series	1	33	F	Canine edentulism	13	1	Osséotite^®^ NT 4.3*13	Conventional drilling TCB	Yes	Coronal	Bone	Two-stage	No	2 – 6	12/12 (100%)	8	12/12 (100%)
		1	85	F	Complete maxillary rehabilitation	13	1	Nanotiteosseotite^®^ 4*10	Conventional drilling	Yes	Radicular	Bone	Two-stage	Yes Mucosa Perforation	2 – 6		5	
		1	72	M	Canine edentulism	13	1	Osséotite^®^ NT 4.3*13	Conventional drilling	Yes	Radicular	Tooth	One-stage	No	2 – 6		3	
		1	64	F	Canine edentulism	23	1	Nobel Active^®^ 4.3*13	Conventional drilling	Yes	Radicular	Bone	One-stage	No	2 – 6		3	
		1	58	M	Canine edentulism	13	1	Nobel Active^®^ 4.3*13	Conventional drilling TCB	Yes	Coronal	Tooth	One-stage	No	2 – 6		2	
		1	32	F	Mobile deciduous canine	13, 23	2	Nobel Active^®^ 4.3*13 Nobel Replace^®^ 4.3*13	Conventional drilling TCB	Yes	1 Coronal 1 Radicular	Bone Tooth	One-stage	No	2 – 6		1.5	
		1	66	F	Partial edentulism	13	2	12: Nobel Active^®^ 3.5*13 13: Nobel Active^®^ 3.5*13	Conventional drilling	Yes	2 Radicular	Bone Bone	One-stage	No	2 – 6		5	
		1	55	F	Mobile deciduous canine	23	1	Nobel Replace^®^ 3.5*15	Conventional drilling TCB	Yes	Coronal	Bone	Two-stage	Yes Soft tissue infection [2 weeks]	2 – 6		1	
		1	69	M	Partial edentulism	23	2	23: Nobel Active^®^ 4.3*13 24: Nobel Active^®^ 4.3*13	Conventional drilling TCB	Yes	1 Coronal 1 Radicular	Tooth Tooth	Two-stage	No			1	
Amato [22] 2019	Case series	7	Ns	Ns	Varied	13, 23, 33, 43	8	3i 3 T and Osséotite^®^, Zimmer Biomet Dental	Piezosurgery Conventional drilling	Ns	Ns	Ns	One-stage	No	6	7/7 (100%)	5 – 7	7/7 (100%)
Brinkmann [23] 2020	Case report	1	78	F	Partial edentulism	33	1	S.I.N. Implant System 3.75*10	Conventional drilling	No	Coronal	Tooth	Two-stage	No	4.5	1/1 (100%)	2	1/1 (100%)
Smojver [24] 2021	Case report	1	55	F	Partial edentulism	13, 23	2	13: Straumann^®^ BLT 4.1 23: Straumann^®^ BLT 4.1	Guided Surgery	Yes	2 Radicular	Bone Tooth	Two-stage	No	3	2/2 (100%)	1	2/2 (100%)
Ouni [25] 2023	Case report	1	22	F	Partial edentulism	33	2	33: Easy Implant^®^ 3.7*11.5 Implant Replacement: Easy Implant^®^ 4*11.5	Conventional drilling	No	Coronal	Ns Ns	Two-stage	Yes Bone loss, exposed threads, mobility after 1 month on the first implant	~ 3	1/2 (50%)	3	1/1 (100%)

*TCB* tungsten carbide bur, *Ns* not specified, *y* years

^a^The same case of the 31-year-old patient was described in both publications

Ankylosis of the impacted maxillary canine is the fusion between the tooth cementum and the surrounding alveolar bone, preventing normal eruption of the tooth. It affects approximately 14.5% of cases, with the incidence increasing with age, reaching 96.8% in individuals over 45. This condition may coexist with external cervical and root resorption, often caused by trauma, infections, or orthodontic treatments [27]. Impacted maxillary canines that are ankylosed may be difficult to remove and can result in significant bone defects. A careful review of 3D X-rays should be performed to identify any loss of continuity in the periodontal space and lamina dura, which would indicate ankylosis [28].

To identify an ankylosed impacted maxillary canine, specific radiological elements are crucial. First, CBCT can reveal the absence of a periodontal space around the tooth, indicating a direct bony contact. Additionally, the presence of a sclerotic bone around the root can suggest ankylosis. Observing a diminished or absent lamina dura is another key indicator, as this layer typically separates the tooth from the surrounding bone. Lastly, a reduced or absent follicular sac surrounding the impacted tooth can signify ankylosis. Together, these radiological findings provide essential information for diagnosing an ankylosed canine [29].

Retrospectively, patient presented preoperatively an external replacement resorption on his impacted canine without clinical symptoms. Thus, drilling trauma and probably bacterial contamination during implant placement induced severe internal inflammatory resorption in few months leading to inflammatory lesion around and infectious complication [18].

In middle-aged patients with an impacted tooth, the conventional standard treatment typically involves extracting the tooth while simultaneously or secondly placing an implant associated to a guided bone regeneration (GBR) [6, 30]. However, this approach is more invasive and can extend treatment time, especially when cortical bone is not preserved, making simultaneous implant placement more difficult [31]. As a result, patients can decline this option. In such cases, placing dental implants through impacted tooth appears to be an alternative for implant-supported restorations [32]. Although long-term results are favorable for placing an implant in contact or through an impacted canine, the total number of cases is very limited and should therefore be interpreted with caution. No implant failures were observed among the 12 implants placed through impacted maxillary canines over a follow-up period of up to 8 years [8]. Additionally, eight implants placed through impacted teeth and immediately loaded in the esthetic zone demonstrated a 100% survival rate at the 5- to 7-year follow-up [22]. A case report demonstrated the design and fabrication of a static guide to assist in bilateral transcanine implant placement. However, only short-term results were provided [24]. It is challenging to deem that is a “valid therapeutic option for implant-supported restorations” without cautioning practitioners about the limited scientific data available; further studies are required [16].

The therapeutic options considered for managing a persistent ankylosis impacted tooth in contact with an implant include several approaches, each with its advantages and disadvantages [5].

In similar cases, complete extraction of the impacted tooth would fully eliminate the source of infection and provides better access for removing the tooth and cyst. However, this option carries significant risks, including creation of a major bone defect, possible communication with the nasal cavity and potential loss of the implant. A new implant could be placed in a second time more or less combined with a GBR. This approach requires a minimum of two separate procedures, with a quite long healing period between each stage.

Coronectomy combined with enucleation of the cystic lesion represents a less invasive solution that preserves the implant while removing the source of infection. This approach limits complications and minimizes bone trauma, though it may require long-term follow-up to ensure no recurrence. The coronectomy of the impacted canine is a conservative and minimally invasive surgical procedure that involves removing only the crown and/or the first third root of the ankylosed impacted tooth while leaving the rest of the root intact. This modality may reduce the potential for risks to the apical regions of the teeth in the surgical field as well as limiting nasal and/or sinus communication [33]. This technique may be typically indicated when a full extraction can induce significant risks, such potential damage to adjacent anatomical structures, which can affect the quality of subsequent prosthetic treatment [5, 34]. In this case, the surgical protocol combined two techniques described in the literature: coronectomy for impacted teeth and decoronation for ankylosed teeth.

The coronectomy procedure is a well-accepted method for managing impacted third molars. It significantly reduces the risk to injure the inferior alveolar nerve (IAN) and minimizes pain and the incidence of localized alveolitis when compared to complete tooth extraction [35, 36]. Bone surgery is performed with a water-cooled burr, allowing for the exposure of the teeth and removes only the crown, leaving the root inside the socket. The root is then smoothed to prevent bone formation around it, ensuring at least 3 mm of root remains below the crest. A radiograph is taken to confirm the complete removal of enamel before suturing [37].

Similarly, a conservative treatment option for ankylosed teeth, known as decoronation, was introduced in 1984 [38]. This technique involves performing a coronectomy on the ankylosed tooth below the level of the cemento-enamel junction, along with instrumentation of the pulp canal to promote bleeding in the periapical region [39]. In post-pubertal patients, the primary goal of decoronation is to preserve the remaining alveolar bone [38]. The main indications for this technique include traumatized, ankylosed, and infraoccluded incisors. Canal instrumentation using endodontic files plays a crucial role in preparing and debriding the root canal, thereby reducing the risks of infection and periradicular inflammation. This procedure also promotes the formation of a blood clot between the residual root and the surrounding bone. Moreover, this exposure of the root to the bone may lead to progressive root resorption. Previous studies have shown that the residual apical fragment is no longer visible 1–10 years after surgery [39]. Therefore, while canal instrumentation is essential for ensuring effective healing, it is imperative to consider its long-term effects on the residual root in the context of clinical management of these cases.

The coronectomy can serve as a valuable therapeutic alternative for managing impacted maxillary canines [33]. Candidates for this technique should be selected with caution and after thorough evaluation. Medically compromised patients with infectious risk, including those who are immunocompromised, diabetic, or on long-term steroid therapy, as well as individuals undergoing cancer treatments like chemotherapy or radiation, are not ideal candidates due to their increased risk of postoperative infections and delayed healing [40]. Local contraindications to this procedure include teeth infection involving the root those associated with recurrent cystic lesions after surgery, and the presence of tumors [41].

## Conclusion

In this report, the coronectomy of the impacted ankylosed canine facilitated a targeted intervention while minimizing surgical trauma. By allowing direct access to the infectious lesion while preserving the integrity of the adjacent implant and preserving bone osteotomy, the procedure ensured a favorable outcome without complications. The successful reossification observed during follow-up underscores the potential of coronectomy may be a viable treatment option in similar cases involving impacted canines.

While coronectomy appears to offer a conservative alternative in managing complex dental scenarios, its inclusion in the therapeutic gradient should be carefully considered on a case-by-case basis, taking into account patient-specific factors and potential long-term outcomes.

## Data Availability

No datasets were generated or analysed during the current study.

## Abbreviations

CBCT: Cone beam computed tomography
GBR: Guided bone regeneration
IAN: Inferior alveolar nerve
